# MSRRT-DETR: A high-precision apple detection method with strong cross-domain generalization capability in complex orchard scenes

**DOI:** 10.1371/journal.pone.0342854

**Published:** 2026-03-13

**Authors:** Xinyu Zhang, Sawut Mamat, Xiaohuang Liu, Jiufen Liu, Run Liu, Guangjie Wu, Ping Zhu, Hongyu Li, Min Ma, Xiaotong Liu

**Affiliations:** 1 College of Geography and Remote Sensing Sciences, Xinjiang University, Urumqi, China; 2 Key Laboratory of Coupling Process and Effect of Natural Resources Elements, Ministry of Natural Resources, Beijing, China; 3 Xinjiang Key Laboratory ossssf Oasis Ecology, Xinjiang University, Urumqi, China; 4 Xinjiang Altay Natural Resources Element Field Observation Research Station, Altay, China; Hohai University, CHINA

## Abstract

Accurate fruit detection is a key component of precision agriculture applications such as crop yield estimation, orchard management, and intelligent harvesting. In scenarios where immature fruits exhibit visual similarity to the background or where significant varietal differences exist, traditional models often lack sufficient generalization ability, resulting in reduced detection accuracy and unstable predictions. To address this problem, this paper proposes a fruit detection model, MSRRT-DETR, which achieves a balance of high accuracy, real-time performance, and strong generalization capability. To improve detection accuracy and robustness in complex orchard environments, MSRRT-DETR introduces three major enhancements to the RT-DETR framework: a Multi-Scale Convolutional Attention Module (MSBlock) to enhance feature representation at different scales; a Spatial and Channel Synergistic Attention Module (SCSA) to improve object focus and discriminative capability; and a Re-parameterized Feature Pyramid Network (RepGFPN) to achieve efficient multi-scale feature fusion. Experimental results show that MSRRT-DETR achieves a mAP50 of 87.3% on the self-constructed TSApple dataset, outperforming mainstream lightweight models YOLOv8, YOLO11, and YOLO12 by 2.0–7.9 percentage points, exceeding two-stage detectors including Faster R-CNN, Mask R-CNN, and Cascade R-CNN by 5.1–8.6 percentage points, and surpassing the RT-DETR series by 1.1–2.6 percentage points. With an inference speed of 30.2 FPS, comparable to the YOLO series, MSRRT-DETR achieves an excellent balance between accuracy and real-time performance. In addition, MSRRT-DETR demonstrates outstanding cross-domain generalization capability on four public datasets including MinneApple, validating its stable applicability across diverse scenarios and fruit varieties. MSRRT-DETR combines high recognition accuracy, fast inference, and strong cross-domain generalization, fully meeting the requirements of fruit detection in complex agricultural scenarios. The model provides robust technical support for intelligent monitoring and automated orchard management in precision agriculture, and holds significant practical value and broad potential for application.

## Introduction

In recent years, deep learning has achieved remarkable progress in agricultural intelligence, particularly demonstrating broad prospects in automated fruit harvesting and yield estimation scenarios. As the fundamental component of these tasks, fruit target detection’s accuracy and robustness directly impact the practicality of agricultural robotic systems [1]. However, in practical orchard environments, fruits often exist at different maturity stages and are susceptible to interference from occlusion, scale variation, and complex lighting conditions. These factors commonly lead to challenges such as small targets and weak boundaries in images, posing significant difficulties for existing detection models [2–4]. Consequently, how to improve target detection models’ precise recognition capability in complex orchard environments while maintaining real-time performance and computational efficiency has become a critical issue in this field.

With the increasing demand for agricultural intelligence, deep learning-based object detection technology has been widely applied in smart agriculture scenarios such as precise fruit recognition and automated harvesting [5–7]. Current mainstream object detection methods mainly include two-stage detectors and one-stage detectors, both of which have been extensively explored and applied in agricultural settings. Among two-stage detectors, Faster R-CNN has attracted attention in agricultural vision due to its high accuracy. Gao et al. [8] used this model to achieve multi-state detection of non-occluded, leaf-occluded and fruit-occluded apples in orchards. However, this method requires generating numerous candidate regions, resulting in high computational complexity and insufficient real-time performance, making it difficult to directly apply in orchard robotic systems.

To address the computational complexity issues of two-stage methods and improve real-time performance, researchers have increasingly focused on one-stage detectors represented by the YOLO series [9–12]. Tian et al. [13] proposed an improved YOLOv3 model for apples at different growth stages, which optimized feature layer propagation through DenseNet architecture and effectively improved detection performance under varying lighting conditions and fruit scale changes. Sun et al. [14] developed ESC-YOLO that uses spatial-channel feature reconstruction convolution (SCConv) to improve detection accuracy under occlusion while maintaining real-time detection speed. However, one-stage models typically employ shared-channel feature pyramids, making it difficult to fully capture fine-grained features of multi-scale fruits, leading to suboptimal performance in dense small-target or heavily occluded scenarios [15]. In recent years, Transformer architectures have provided new research directions for object detection due to their excellent global modeling capabilities [16]. DETR-based models have significantly improved object detection performance in complex environments through attention mechanisms [17]. Hu & Li [18] proposed the FC-DETR model that achieves high recognition accuracy in heavily occluded and noise-interfered environments through cross-scale adaptive feature fusion and efficient loss function design. However, standard Transformers exhibit high computational complexity when processing high-resolution images, making them difficult to run efficiently on resource-constrained edge devices, thus limiting their practical deployment in real-time orchard detection tasks [19].

To address the aforementioned challenges, this paper proposes MSRRT-DETR, an enhanced fruit detection model built upon the RT-DETR framework with three key improvements. First, a Multi-Scale Convolutional Attention Module (MSBlock) is designed to improve feature representation across different scales and enhance the receptive field adaptability for fruit targets. Second, a Spatial and Channel Collaborative Attention Module (SCSA) is introduced, which integrates spatial multi-semantic attention with progressive channel self-attention to improve the model’s sensitivity to occluded regions, small objects, and complex backgrounds, thereby enhancing both focus and discriminative precision. Third, to overcome performance bottlenecks in feature fusion, a Re-parameterized Feature Pyramid Network (RepGFPN) is incorporated, leveraging non-shared channels and multi-scale interaction to achieve more efficient feature aggregation and representation. The proposed model adopts an end-to-end architecture and employs the Complete IoU loss function to further optimize localization accuracy.

Experimental results demonstrate that MSRRT-DETR outperforms all compared methods across key metrics including mAP50, mAP50:90, and precision, with notable improvements in detecting small and occluded targets. Compared with YOLO series models, MSRRT-DETR achieves a 2.0%–7.9% gain in mAP50 while maintaining comparable inference speed (30.2 FPS), enabling real-time detection. Relative to two-stage detectors, it achieves significantly higher accuracy and inference speed with nearly half the number of parameters. Compared with its RT-DETR counterparts, MSRRT-DETR offers a moderate improvement in accuracy while reducing parameter count by approximately one-third and doubling the FPS. Moreover, MSRRT-DETR demonstrates superior cross-domain generalization on four unseen public fruit datasets including MinneApple, consistently ranking first or second across all major metrics, validating its robustness and adaptability in diverse orchard environments.

The main contributions of this paper are as follows:

1. A high-quality apple detection dataset (TSAppleData) is constructed, covering various fruit growth stages, occlusion types, scale variations, and complex lighting conditions, providing a solid benchmark for fruit detection in challenging orchard scenarios.
2. An effective transformer-based detection network, MSRRT-DETR, is proposed, which integrates multi-scale feature construction, spatial-channel collaborative attention, and efficient feature fusion to significantly improve detection precision and robustness, particularly for small or occluded targets.
3. Extensive experiments on both the proposed dataset and multiple public datasets validate that MSRRT-DETR achieves a well-balanced performance in accuracy, speed, and generalization, consistently outperforming YOLO series, two-stage detectors, and baseline RT-DETR models.

## Construction of TSAppleData dataset and data augmentation

### Data collection

**Fig 1:**
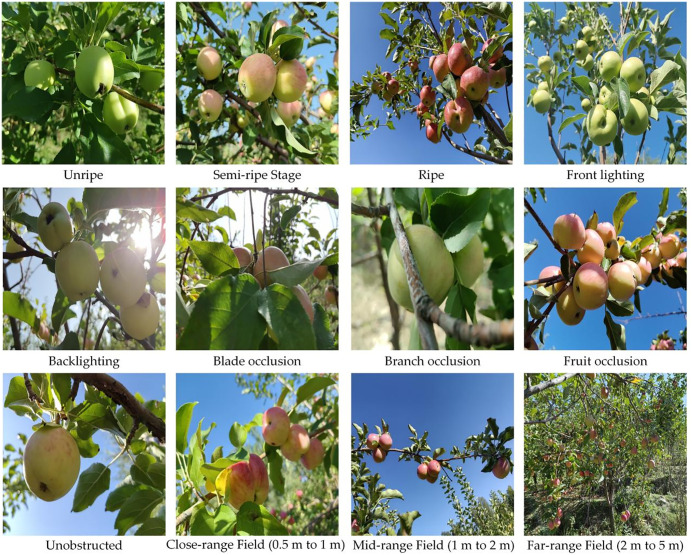
Sample images from TSAppleData Dataset, showing various growth stages, lighting conditions, occlusion types, and shooting distances.

From July to September 2024, we conducted multiple field surveys in apple orchards in Shuimogou District, Urumqi, Xinjiang, China, collecting an apple image dataset named TSAppleData Dataset that covers different temporal phases and spatial distributions. The temporal dimension included various growth stages and harvesting times within a day, while the spatial dimension covered different occlusion conditions and shooting distances. Using a HUAWEI nova 12 smartphone, we captured 1,266 apple images (3000 × 4000 resolution). After manual screening and downsampling, we obtained 1,078 high-quality images (640 × 640 resolution). Fig 1 displays a selection of images from the TSAppleData Datasets.

Data collection spanned three maturity stages (immature, semi-mature, mature) to ensure comprehensive growth stage coverage. We scheduled collection between 11:00–14:00 and 17:00–19:00 to account for lighting variations, maintaining balanced frontlight/backlight representation.

For spatial distribution, we categorized occlusion into four types: leaf occlusion, branch occlusion, fruit-to-fruit occlusion, and non-occlusion [8]. Images were captured at close (0.5-1m), medium (1-2m), and long (2-5m) distances to provide multi-scale spatial information.

### Data annotation and dataset partitioning

This study employed LabelImg software for precise annotation of apple images. During annotation, the bounding box size reflected the relative distance between fruit targets and the camera. Considering the limited coverage range of robotic arms, distant small targets on different branches within the same image as close-range targets were treated as background and not annotated, thereby reducing false detections and performance waste [20]. After annotation, the dataset was randomly divided into training, validation, and test sets at a ratio of 7:2:1 to ensure scientific rigor and effectiveness in model training, validation, and testing.

### Data augmentation

**Fig 2:**
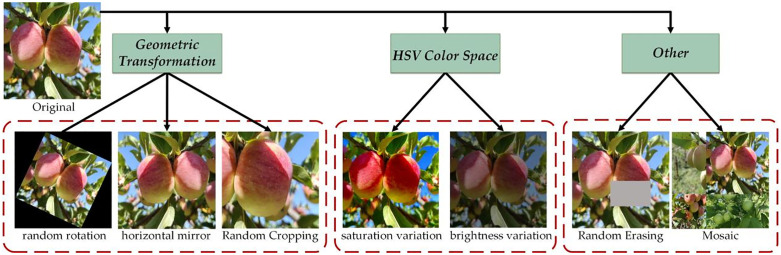
Schematic diagram of partial data augmentation methods for Online Augmentation.

To enhance dataset diversity, improve model robustness, and effectively prevent overfitting, this study applied online augmentation techniques to process the apple image dataset. Specifically, the augmentation operations included comprehensive geometric transformations such as random rotation, translation, scaling, horizontal flipping, vertical flipping, and cropping to simulate apples’ appearance from various perspectives and positions. Additionally, adjustments to hue, saturation, and brightness in HSV color space were implemented to replicate color variations under different lighting conditions. To further strengthen the model’s robustness against practical shooting factors like occlusion, overlap, and object interactions in complex scenarios, advanced augmentation methods including Random Erasing, Mosaic, and Mixup were introduced. These techniques improved dataset diversity and enhanced the model’s adaptability to environmental disturbances. Detailed illustrations of the data augmentation methods are shown in Fig 2.

## Method overview

### Overall architecture of MSRRT-DETR

**Fig 3:**
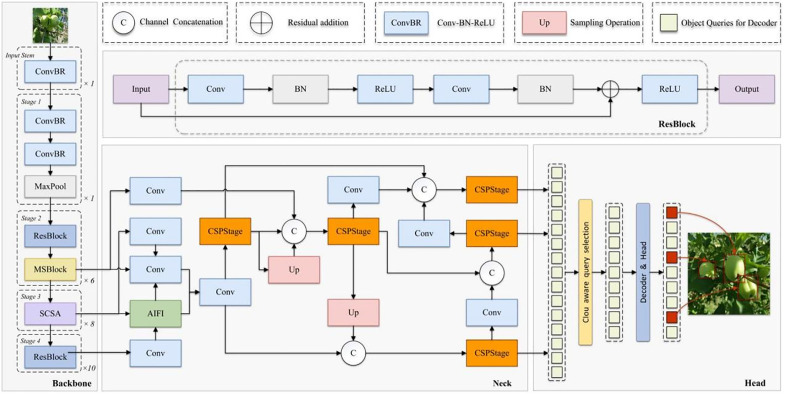
Schematic diagram of the MSRRT-DETR overall architecture, where ResBlock denotes the residual module.

Existing detection models still face significant challenges in accurately identifying and localizing occluded targets in complex real-world environments with diverse objects and frequent occlusions. To address this, we propose an optimized object detection model based on the RT-DETR architecture [21], as illustrated in Fig 3. The model employs a modified lightweight ResNet as backbone network for efficient feature extraction. A Spatial and Channel Reconstruction Convolution module (MSBlock) is introduced to enhance multi-scale spatial representation. The Spatial and Channel Collaborative Attention mechanism (SCSA) enables joint modeling of spatial-channel information.

For the neck network, we implement RepGFPN structure with non-shared channel configuration for dense target representation. Complete IoU (CIoU) loss function is adopted for precise bounding box optimization.

### Improved backbone feature extraction network

#### Lightweight ResNet network.

**Fig 4:**
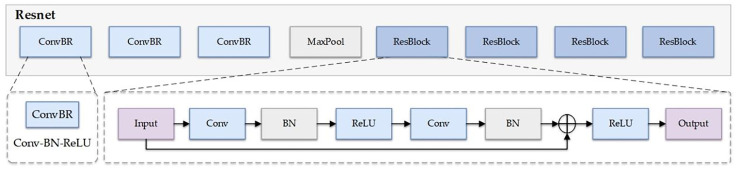
Schematic diagram of ResNet architecture.

The backbone network of the RT-DETR model employs a lightweight ResNet architecture, achieving efficient feature extraction through streamlined module design and channel optimization [22], with its structure shown in Fig 4. The network primarily consists of three components: an initial downsampling stage, a deep residual extraction stage, and a multi-scale output module. The initial stage sequentially applies three ConvNormLayer operations and one max pooling operation to accomplish feature space compression and low-level feature capture. Subsequently, four sets of residual blocks (Blocks) extract mid-to-high level semantic information with 64, 128, 256, and 512 channels respectively, outputting feature maps at three scales (P3, P4, P5) for subsequent detection heads to perform multi-scale target modeling. Each Block consists of multiple BasicBlocks, exhibiting excellent feature reusability and computational efficiency to meet the representation requirements for complex targets and multi-scale objects in object detection tasks. While maintaining the model’s overall inference speed, this backbone network provides high-quality semantic features for subsequent feature fusion and decoder modules.

#### Multi-scale Feature Construction Module.

**Fig 5:**
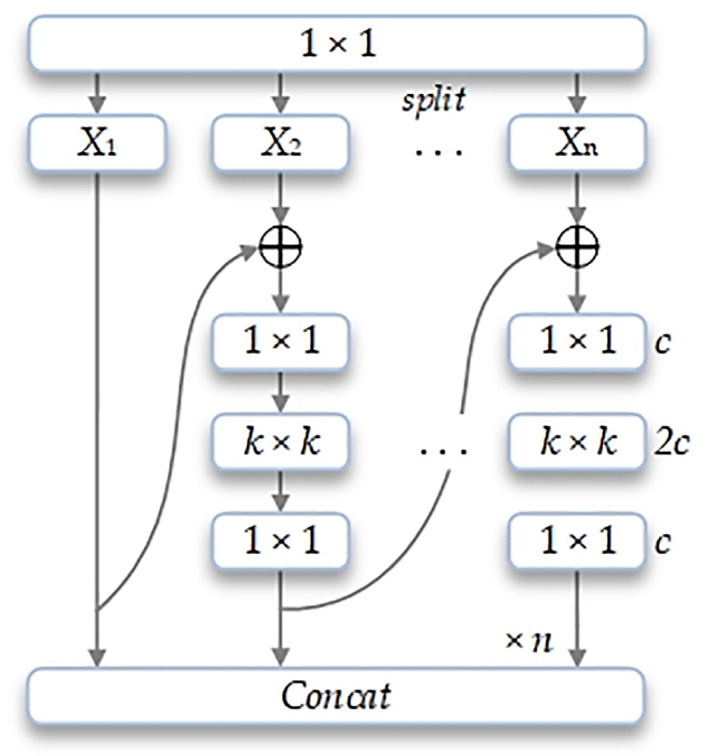
Schematic diagram of MSBlock architecture.

The MSBlock primarily consists of three components: channel expansion and grouping, inverted bottleneck branches, and feature fusion modules. This structure draws inspiration from Res2Net’s hierarchical feature fusion concept while incorporating large-kernel inverted bottleneck architectures to achieve efficient and comprehensive multi-scale feature representation [23,24]. The schematic diagram of the MSBlock structure is shown in Fig 5.

First, the input feature $\text{X}\in \;{\text{R}}^{\text{H }\times \text{W }\times \text{ C}}$ undergoes channel expansion via 1×1 convolution to obtain feature maps with n ×C channels. The expanded features are then evenly divided into n groups along the channel dimension, denoted as ${\text{X}}_{\text{1}},\;{\text{X}}_{\text{2}},\;{\text{X}}_{\text{n}}$. To achieve an optimal balance between representational capacity and computational efficiency, this study sets n = 3.

Building upon the feature grouping, MSBlock employs a progressive fusion strategy to process each group’s features. The first group serves directly as a cross-stage connection, preserving the original feature information. The remaining groups (e.g., ${\text{X}}_{\text{2}},{\text{X}}_{\text{3}}$) undergo fusion with the output from the previous group through inverted bottleneck branches (Inverted Bottleneck, $\text{I}{\text{B}}_{\text{k}\times \text{k}}$), achieving hierarchical extraction of multi-scale features. The specific operations are as follows:


$${\text{Y}}_{\text{i}}=\left\{ \;\;\;\begin{array}{c} {\text{X}}_{\text{i}},\;\;\;\;\;\;\;\;\;\;\;\text{i}=\text{1}\\ \text{I}{\text{B}}_{\text{k}\times \text{k}}\left({\text{Y}}_{\text{i}-\text{1}}+{\text{X}}_{\text{i}}\right).\;\text{i}>\text{1} \end{array}\right.\;$$ (1)

$\text{I}{\text{B}}_{\text{k}\times \text{k}}\left(.\right)$ denotes an inverted bottleneck structure equipped with large convolutional kernels, where k represents the kernel size. This design facilitates effective multi-scale information interaction by integrating the output from the previous group with the input of the current group.

The outputs from all branches, denoted as 1 ×1, are concatenated along the channel dimension and subsequently processed by a ${\text{Y}}_{\text{i}}$ convolution to enable global information exchange and channel-wise feature recalibration. This operation ensures that the fused features are well adapted to the subsequent network structure.

The proposed MSBlock not only enhances the backbone network’s capability to capture and exploit multi-scale features, but also maintains a favorable balance between computational cost and inference speed. The enriched grouping and fusion mechanisms significantly improve the network’s robustness and detection accuracy when dealing with objects of varying scales and diverse scene complexities.

#### Spatial and channel synergistic attention mechanism.

The Spatial and Channel Synergistic Attention (SCSA) mechanism focuses on the joint optimization of spatial and channel information. It integrates two key components—Shared Multi-Semantic Spatial Attention (SMSA) and Progressive Channel Self-Attention (PCSA)—to fully exploit the representational potential of features along both spatial and channel dimensions through a cascaded design [25,26].

In the SMSA module, the input feature $\text{X}\in {\text{R}}^{\text{B}\times \text{C}\times \text{H}\times \text{W}}$ is first subjected to global average pooling along the spatial dimensions, producing two separate feature sequences corresponding to the height and width directions, respectively:


$${\text{X}}_{\text{H}}={\text{AvgPool}}_{\text{H}}\left(\text{X}\right),\;{\text{X}}_{\text{W}}={\text{AvgPool}}_{\text{W}}\left(\text{X}\right)$$ (2)

Subsequently, ${\text{ X}}_{\text{H }}$ and ${\text{ X}}_{\text{W}}$ are partitioned along the channel dimension into K equally sized and mutually independent sub-features, denoted as ${\text{X}}_{\text{H}}^{\text{ i}}$ and ${\text{X}}_{\text{W}}^{\text{ i}}$, respectively. Each sub-feature is then processed using depthwise separable one-dimensional convolutions with distinct kernel sizes, enabling the efficient capture of multi-semantic structural information distributed across different spatial regions.


$${\text{X}}_{\text{H}}^{\text{i}}=\text{DWConv1}{\text{d}}_{\text{C}/\text{K}\to \text{C}/\text{K}}^{{\text{k}}_{\text{i}}}\left({\text{X}}_{\text{H}}^{\text{i}}\right),\;{\text{X}}_{\text{W}}^{\text{i}}=\text{DWConv1}{\text{d}}_{\text{C}/\text{K}\to \text{C}/\text{K}}^{{\text{k}}_{\text{i}}}\left({\text{X}}_{\text{W}}^{\text{i}}\right)$$ (3)

The outputs from the above branches are concatenated along the channel dimension and normalized using Group Normalization, which effectively mitigates the semantic interference commonly introduced by Batch Normalization. A Sigmoid activation is then applied to generate the final spatial attention map.


$$\text{Att}{\text{n}}_{\text{H}}=\sigma \left({\text{GN}}_{\text{K}}\left(\text{Concat}\left({\text{X}}_{\text{H}}^{\text{1}},...,{\text{X}}_{\text{H}}^{\text{K}}\right)\right)\right),\;\text{Att}{\text{n}}_{\text{W}}=\sigma \left({\text{GN}}_{\text{K}}\left(\text{Concat}\left({\text{X}}_{\text{W}}^{\text{1}},...,{\text{X}}_{\text{W}}^{\text{K}}\right)\right)\right)$$ (4)

The spatial attention map performs element-wise weighting on the original input features, enhancing multi-scale spatial information and ultimately yielding a spatially restructured feature representation.


$${\text{X}}_{\text{s}}=\text{Att}{\text{n}}_{\text{H}}\times \text{Att}{\text{n}}_{\text{W}}\times \text{X}$$ (5)

Building upon this, the PCSA module further performs channel dependency modeling on the spatially enhanced features ${\text{X}}_{\text{s}}$. First, ${\text{X}}_{\text{s}}$ undergoes spatial dimension reduction via pooling to reduce computational cost, followed by depthwise separable convolution for linear projection, producing the query (Q), key (K), and value V()vectors along the channel dimension:


$$\text{Q}={\text{F}}_{\text{proj}}^{\text{Q}}\left({\text{X}}_{\text{p}}\right),\;\text{K}={\text{F}}_{\text{proj}}^{\text{K}}\left({\text{X}}_{\text{p}}\right),\;\text{V}={\text{F}}_{\text{proj}}^{\text{V}}\left({\text{X}}_{\text{p}}\right)$$ (6)

A single-head self-attention mechanism is then applied along the channel dimension to compute global inter-channel correlations:


$${\text{X}}_{\text{attn}}=\text{Softmax}\left(\frac{\text{Q}{\text{K}}^{\text{T}}}{\sqrt{\text{C}}}\right)\text{V}$$ (7)

Subsequently, FFF is passed through global pooling and a Sigmoid activation function to generate channel attention weights, which are used to reweight the spatially enhanced features across the channel dimension, thereby achieving synergistic fusion of spatial and channel information:


$${\text{X}}_{\text{c}}={\text{X}}_{\text{s}}\times \sigma \left({\text{Pool}}_{\left({\text{H}}^{\prime },{\text{W}}^{\prime }\right)\to \left(\text{1},\text{1}\right)}\left({\text{X}}_{\text{attn}}\right)\right)$$ (8)

**Fig 6:**
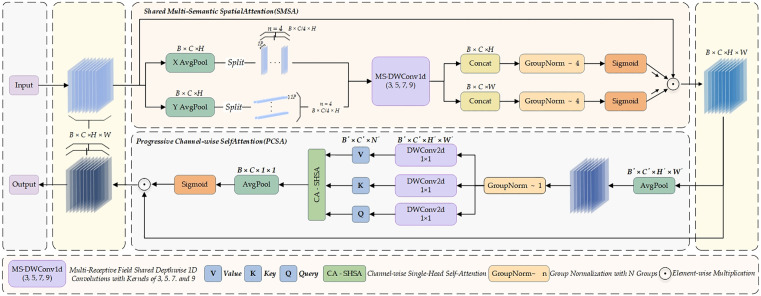
Schematic diagram of the SCSA attention mechanism structure.

The design of SCSA fully considers the interaction and complementarity between spatial and channel information. The SMSA module leverages a multi-branch, multi-scale grouped convolution mechanism to effectively enhance the model’s ability to represent different levels of spatial semantics, enabling precise emphasis on salient regions while suppressing redundant noise. On top of spatial enhancement, the PCSA module employs a self-attention mechanism to model global channel dependencies, allowing for fine-grained modeling of high-level channel semantics. The organic integration of these two modules significantly alleviates the semantic fragmentation commonly caused by the decoupling of spatial and channel attention in conventional mechanisms, thereby enhancing the diversity and robustness of feature representations.The overall architecture of SCSA is illustrated in Fig 6.

### Neck with reparameterized generalized feature pyramid network

**Fig 7:**
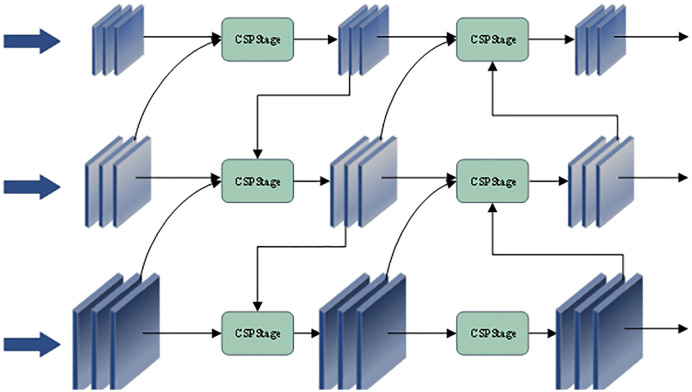
Schematic illustration of the Efficient RepGFPN architecture.

To enhance the model’s multi-scale feature fusion capability, this work adopts an efficient Reparameterized Generalized Feature Pyramid Network (RepGFPN) as the neck structure [27], whose overall architecture is illustrated in Fig 7. Building upon the classical Feature Pyramid Network (FPN), RepGFPN incorporates several key techniques, including a non-shared channel configuration, a re-parameterization mechanism, as well as CSP structures and ELAN-style connections, effectively mitigating the limitations of the traditional FPN architecture [28].

Unlike the standard FPN, which enforces channel sharing across features at different scales, RepGFPN employs a non-shared channel configuration. This allows feature maps at each specific scale to retain their native spatial and channel characteristics. This design eliminates the need for the frequent rescaling and dimensional alignment operations required in traditional methods, thereby improving the efficiency of multi-scale feature fusion while simultaneously enhancing feature representational capacity.

**Fig 8:**
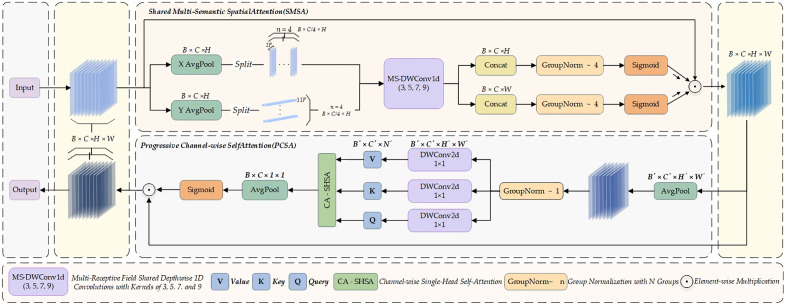
Structural illustration of the CSPStage and Rep modules.

In addition to optimizing the fusion process, RepGFPN introduces an enhanced cross-scale feature interaction mechanism. At its core, it integrates the CSPStage and Rep modules (as shown in Fig 8) within the neck structure. Specifically, five CSPStage modules are employed to incorporate multi-scale feature inputs from both adjacent and same-level layers, enabling more comprehensive feature fusion. This design significantly promotes the flow and interaction of multi-scale information across feature layers, thereby enhancing feature reuse and representation power.

Moreover, this mechanism achieves these improvements without imposing substantial additional computational overhead, leading to higher detection accuracy and robustness. As a result, RepGFPN provides a strong technical foundation for object detection tasks under complex real-world scenarios.

### Complete IoU loss function

To enhance the performance of bounding box regression in object detection, this work adopts the Complete Intersection over Union (CIoU) loss function. Unlike conventional IoU-based losses that consider only the overlap area between the predicted and ground truth boxes, CIoU additionally takes into account the distance between box centers and the aspect ratio, thereby providing a more comprehensive and effective optimization of the regression targets [29].

Specifically, let the predicted bounding box be denoted as B, and the ground truth box as ${\text{B}}_{\text{gt}}$. The CIoU loss is defined as follows:


$${\text{L}}_{\text{CIoU}}=\text{1}-\text{IoU}+\frac{\rho^{\text{2}}\left(\text{b},{\text{b}}_{\text{gt}}\right)}{{\text{c}}^{\text{2}}}+\alpha \text{v}$$ (9)

where IoU represents the intersection-over-union between the predicted and ground truth boxes. $\rho \left(\text{b},{\text{b}}_{\text{gt}}\right)$ denotes the Euclidean distance between the centers of the predicted and ground truth boxes. c is the diagonal length of the smallest enclosing box covering both bounding boxes. The term v measures the consistency of aspect ratios and is defined as:


$$\text{v}=\frac{\text{4}}{\pi^{\text{2}}}{\left(\text{arctan}\frac{{\text{w}}_{\text{gt}}}{{\text{h}}_{\text{gt}}}-\text{arctan}\frac{\text{w}}{\text{h}}\right)}^{\text{2}}$$ (10)

α is a weighting factor for the regularization term, computed as:


$$\alpha =\frac{\text{v}}{\left(\text{1}-\text{IoU}\right)+\text{v}}$$ (11)

where (w,h) and $\left({\text{w}}_{\text{gt}},{\text{h}}_{\text{gt}}\right)$ correspond to the widths and heights of the predicted and ground truth boxes, respectively.

Unlike traditional IoU-based loss functions, the CIoU loss simultaneously optimizes localization accuracy and shape constraints, which facilitates faster convergence and improves the final detection performance.

## Experimental results and analysis

### Ablation study on module design

To evaluate the impact of each improved module on overall performance, we conducted multiple ablation experiments by progressively introducing the multi-scale feature extraction module (MSBlock), spatial-channel collaborative attention mechanism (SCSA), and efficient feature fusion module (RepGFPN). The changes in detection accuracy, inference efficiency, and computational cost under different configurations were analyzed. The experimental results are shown in Table 1.

**Table 1:** Ablation Study on the TSAppleData Dataset.

Method	MSBlock	SCSA	RepGFPN	mAP50/%	mAP50:90/%	Precision/%	Recall/%	Params/M	FPS	GFLOPs
1				84.7 ± 0.43*	68.6 ± 1.39*	85.9 ± 1.14*	76.2 ± 1.74*	20.09	26.7	58.3
2	√			84.4 ± 0.42*	67.4 ± 0.19*	87.1 ± 1.00	77.3 ± 1.16	20.25	37.1	59.7
3	√	√		86.1 ± 0.43*	70.3 ± 0.40*	85.5 ± 1.37*	79.4 ± 0.94*	20.18	22.3	59.7
4	√	√	√	**87.3 ± 0.45**	**73.0 ± 0.80**	**88.2 ± 1.10**	**80.3 ± 0.49**	24.95	**30.2**	66.3

Note: Method 1 denotes the baseline RT-DETR-18 model. Method 2: RT-DETR-18 + MSBlock. Method 3: RT-DETR-18 + MSBlock + SCSA. Method 4: RT-DETR-18 + MSBlock + SCSA + RepGFPN (i.e., the proposed MSRRT-DETR). * indicates a statistically significant difference compared to the proposed MSRRT-DETR (p < 0.05).

As shown in Table 1, after introducing MSBlock (Method 2) to the baseline model (Method 1), the inference speed increased from 26.7 FPS to 37.1 FPS, representing a 39% improvement, while GFLOPs only increased by 2.4%. Meanwhile, Precision improved to 87.1% and Recall increased to 77.3%. These results indicate that MSBlock accelerates inference with minimal additional computational cost and positively impacts classification performance.

By adding the SCSA module to Method 2 (Method 3), the model’s mAP50 improved from 84.4% to 86.1%, mAP50:90 increased from 67.4% to 70.3%, and Recall further improved to 79.4%, indicating significant enhancement in feature representation capability and target localization accuracy. Although SCSA introduced additional computational overhead, reducing inference speed to 22.3 FPS, it substantially improved detection performance in complex scenarios [30]. The enhanced feature representation and localization accuracy provide superior comprehensive performance, which is particularly valuable for applications requiring high detection precision.

Further integrating RepGFPN into Method 3 (Method 4) achieved optimal overall performance. mAP50 increased to 87.3%, mAP50:90 improved to 73.0%, Precision reached 88.2%, and Recall rose to 80.3%. Inference speed also recovered from 22.3 FPS to 30.2 FPS. This demonstrates that RepGFPN successfully mitigated the inference speed reduction caused by the attention mechanism while strengthening multi-scale feature fusion and enriching local detail representation. Although parameter count increased, the performance gains far outweighed the additional computational cost in real-time detection and complex orchard environments.

The ablation study results show that MSBlock, SCSA and RepGFPN effectively complement different performance dimensions. MSBlock significantly accelerates inference while improving precision, SCSA enhances feature representation and target localization, and RepGFPN optimizes the balance between inference efficiency and detection accuracy while strengthening feature fusion. Through their synergistic effects, the improved RT-DETR model achieved simultaneous improvements in detection accuracy, recall rate and inference speed, demonstrating particularly strong comprehensive performance in complex scenarios involving weak features and small object recognition. Statistical tests confirm that our method significantly outperforms all other configurations in terms of comprehensive metrics including mAP50 and mAP50:90 with a p-value less than 0.01. Furthermore, it achieves statistically significant improvements in Precision compared to the baseline and Method 3 and in Recall compared to the baseline and Method 2.

### Attention mechanism comparison experiments

To systematically evaluate the impact of different attention mechanism modules on the improved model’s performance, this study adopted a unified baseline network architecture and implemented configurations including no attention mechanism (No Attention) or replacement with current mainstream attention mechanism modules, namely SENetV1, EMA, Biformer, and the proposed SCSA module. The experimental results are shown in Table 2.

**Table 2:** Performance comparison of different attention mechanisms on MSRRT-DETR evaluated on the TSAppleData dataset.

Attention module	mAP50/%	mAP50:90/%	Precision/%	Recall/%	Params/M	FPS
**NO Attention**	86.3 ± 0.53*	70.9 ± 0.70*	86.9 ± 0.58	80.2 ± 0.47	**24.88**	**31.4**
**+SENetV1**	87.2 ± 0.50	71.6 ± 0.18*	88.2 ± 0.75	81.2 ± 0.97	25.05	27.6
**+EMA**	87.1 ± 0.24	71.2 ± 0.19*	**88.5 ± 0.73**	81.6 ± 0.74*	24.99	24.9
**+Biformer**	86.5 ± 0.40*	70.0 ± 0.30*	87.3 ± 1.06	79.8 ± 0.58	25.59	22.7
**+SCSA(ours)**	**87.3 ± 0.45**	**73.0 ± 0.80**	88.2 ± 1.10	80.3 ± 0.49	24.95	30.2

Note: * indicates a statistically significant difference compared to the proposed MSRRT-DETR (p < 0.05).

The experiments on attention mechanism integration indicate that embedding attention modules can improve the model’s detection performance to varying degrees while keeping the computational cost largely unchanged; however, their impact on the trade-off between inference efficiency and accuracy varies significantly. EMA demonstrates strong performance in improving recognition accuracy and recall rate, but also leads to a significant decrease in model inference speed. In contrast, the SCSA module achieves the best mAP50:90 of 73.0% while maintaining high recognition performance and an inference speed second only to the no-attention baseline. The t-test results further confirm its superiority in high-precision detection scenarios, showing significantly better performance than other state-of-the-art attention methods. For metrics that did not reach statistical significance, the performance of the competing attention modules is essentially on par with SCSA, indicating that the advantages of the SCSA module are mainly manifested in high-precision detection scenarios rather than showing significant improvements across all evaluation metrics.

**Fig 9:**
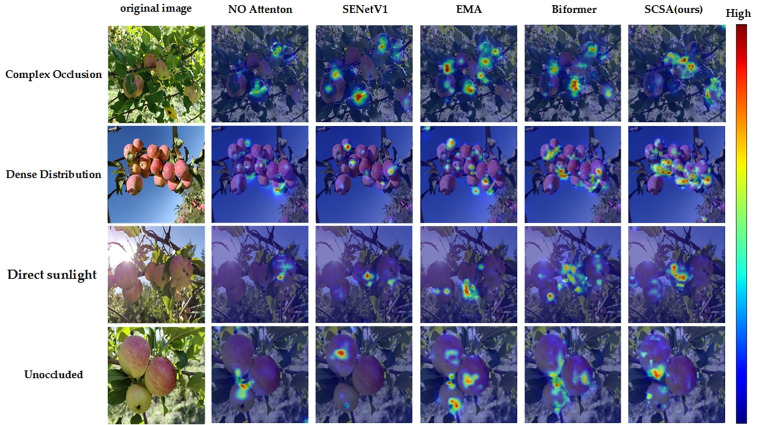
Feature response heatmaps of different attention mechanisms in apple detection across multiple scenarios.

As shown in Fig 9, The heatmap comparison results further demonstrate that attention mechanisms play a significant role in guiding feature extraction towards key regions, with distinct differences observed in feature response locations across various mechanisms. The no-attention mechanism (No Attention) model exhibits dispersed attention and frequently misses critical target areas when confronted with complex backgrounds and occlusion scenarios. While SENetV1 and EMA mechanisms show more concentrated focus on targets, they also display noticeable erroneous activation in background interference regions – for instance, the EMA mechanism incorrectly activates attention on branches in the upper-left corner of densely distributed images. The Biformer mechanism demonstrates relatively scattered attention distribution with insufficient boundary awareness.

In contrast, our proposed SCSA mechanism more effectively concentrates attention on target regions, accurately capturing target contours under complex occlusion and background interference while effectively suppressing attention responses to non-target areas. Moreover, compared to other attention mechanisms, SCSA demonstrates superior performance in small target recognition. Further analysis reveals that SCSA possesses stronger boundary perception and regional discrimination capabilities at the feature level, particularly exhibiting more stable performance when processing small-sized, highly overlapping, or occluded targets. Its achievement of 73.0% on the mAP50:90 metric significantly surpasses other attention mechanisms, quantitatively validating its advantages in target localization accuracy.

### Detection performance analysis across different complex scenarios

**Fig 10:**
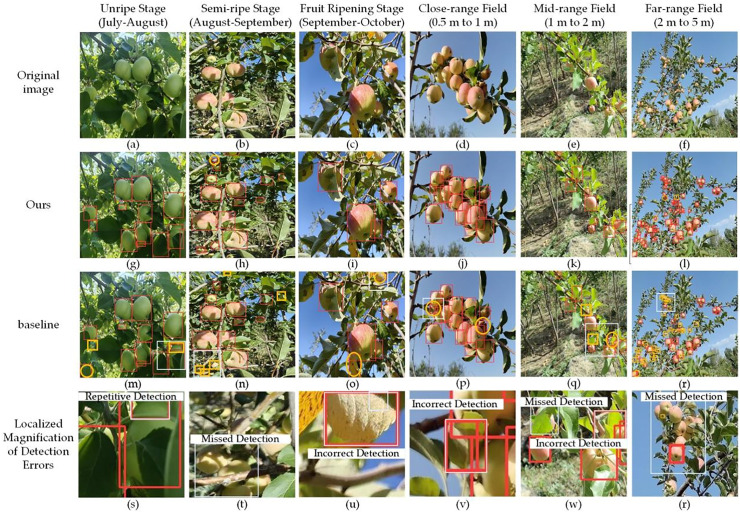
Comparison of detection results before and after model improvement for apples at different growth stages and spatial distributions, along with typical false detection examples. The first row shows original images, the second and third rows display inference results from the improved and original models respectively, while the fourth row presents enlarged views of error regions from the original model (marked by gray boxes). Undetected targets are indicated by orange boxes, and false detections by orange circles.

To comprehensively evaluate the detection performance of the MSRRT-DETR model in complex natural scenarios, this study conducted systematic analysis from both temporal and spatial dimensions. Temporally, three growth stages of apples were selected for investigation: immature, semi-mature, and mature. Spatially, three viewing fields were examined: close-range, medium-range, and long-range. Since all images were captured in natural environments and randomly contained various scenarios including front lighting, back lighting, and fruit occlusion, these characteristics existed as natural attributes in each image and were therefore not listed as separate scenarios. As shown in Fig 10, the improved model demonstrated significantly superior detection performance over the original model across different temporal stages and spatial distribution conditions.

In the temporal dimension, fruits at immature and semi-mature stages mostly appear green or light red, exhibiting high color similarity with background leaves and constituting typical low-contrast detection scenarios. In such images, the original model failed to effectively distinguish fruit from background information and showed deviations in boundary recognition at fruit overlapping regions, resulting in relatively obvious missed and false detection issues as shown in Figure m. In contrast, the detection results of the improved model under the same scenarios (as shown in Figure g) could more accurately distinguish detection targets from image backgrounds, reflecting its stronger perception and representation capabilities in low-contrast and weak-edge regions.

In the spatial dimension, as the shooting field of view moved from near to far, the pixel size of fruit targets continuously decreased while detail information gradually reduced, particularly under medium-to-long range viewing conditions, which placed higher demands on the model’s scale perception and multi-scale feature fusion capabilities. In such scenarios, the original model showed significantly increased missed detection rates and markedly degraded detection performance. As shown in Figure r, numerous problems including repeated detections, missed detections and false detections appeared in areas with dense targets or severe occlusion, revealing its insufficient robustness under complex conditions. Compared with the original model, the improved model demonstrated significant performance advantages in complex scenarios, especially for small-size target detection. Under the long-range viewing fields shown in Figures l and r, MSRRT-DETR exhibited stronger small-target detection capability compared to the original model, further demonstrating its superior scale robustness.

Further analysis of typical detection errors revealed in Figures s-t shows that the original model had multiple performance deficiencies in complex scenarios. For instance, in areas with complicated lighting conditions such as backlighting or bright backgrounds (shown in Figures o and p), the original model tended to falsely detect bright areas as apples (detailed in Figures u and v), or miss real targets in shadowed areas (shown in Figure t). When targets were severely occluded, it struggled to accurately infer the contours of occluded targets, leading to missed or false detections of fruits as shown in Figure w. In comparison, the improved model demonstrated detection performance significantly superior to the original model in backlit and bright background areas (shown in Figures i and j), while also achieving more accurate recognition and localization of fruit targets under shadowed and severely occluded conditions (shown in Figure k). This performance improvement mainly benefits from multi-level optimization of the model’s feature extraction and fusion strategies. Specifically, the improved model introduced a multi-scale perception module at shallow layers, incorporated spatial-channel attention mechanisms at middle layers, and reconstructed multi-scale feature transmission paths at detection heads, thereby effectively enhancing the model’s perception capability for feature regions and target discrimination ability in complex scenarios. Consequently, under adverse factors like strong light interference and shadow occlusion, the improved model could more stably focus on effective target areas, overall exhibiting higher detection robustness and better environmental adaptability.

### Performance comparative analysis with mainstream detectors

To thoroughly investigate the comprehensive performance of the improved MSRRT-DETR model in object detection tasks, this paper conducted a systematic comparative analysis with current mainstream object detection models, with the results presented in Table 3.

**Table 3:** Performance comparison of MSRRT-DETR versus mainstream object detection models on the TSAppleData dataset.

methods	mAP50/%	mAP50:90/%	Precision/%	Recall/%	Params/M	FPS	GFLOPs
YOLOv8	79.4 ± 0.51*	60.9 ± 0.18*	84.6 ± 1.00*	69.9 ± 1.55*	3.01	30.7	8.1
YOLO11	85.3 ± 0.27*	67.3 ± 0.19*	85.9 ± 1.66*	77.5 ± 1.16*	2.58	**31.1**	6.3
YOLO12	82.9 ± 0.40*	64.0 ± 0.32*	83.9 ± 1.37*	75.0 ± 1.03*	**2.52**	28.3	6.0
Faster-R-CNN	82.2 ± 0.26*	65.7 ± 0.19*	66.9 ± 3.83*	**86.2** ± 0.48*	41.3	6.6	133.9
Mask-R-CNN	79.1 ± 0.57*	60.5 ± 0.38*	74.3 ± 4.73*	82.3 ± 0.75*	30.9	5.7	101.2
Cascsde-R-CNN	78.7 ± 0.27*	59.5 ± 0.26*	75.9 ± 1.71*	82.2 ± 0.37*	57.12	2.1	546.8
RT-DETR-L	86.2 ± 0.53*	69.3 ± 0.70*	85.8 ± 1.18*	79.2 ± 0.94	33.53	22.6	109.4
RT-DETR-18	84.7 ± 0.43*	68.6 ± 1.39*	85.9 ± 1.14*	76.2 ± 1.74*	20.09	26.7	58.3
RT-DETR-50	85.8 ± 0.62*	70.4 ± 0.32*	85.2 ± 0.75*	78.3 ± 0.97*	42.97	15	134.8
MSRRT-DETR	**87.3** ± 0.45	**73.0** ± 0.80	**88.2** ± 1.10	80.3 ± 0.49	24.95	30.2	66.3

Note: * indicates a statistically significant difference compared to the proposed MSRRT-DETR (p < 0.05).

As shown in Table 3, compared to lightweight YOLO series models, the proposed model demonstrates significant advantages across all accuracy metrics. Particularly for mAP50:90, it achieves improvements of 12.1%, 5.7%, and 9.0% over YOLOv8, YOLO11, and YOLO12 respectively. With an inference speed of 30.2 FPS, comparable to the YOLO series, the model maintains excellent real-time performance while significantly enhancing high-precision target recognition capabilities.

Compared to two-stage detectors (Faster R-CNN, Mask R-CNN, Cascade R-CNN), MSRRT-DETR achieves comprehensive superiority in both accuracy and efficiency. It shows improvements of 7.3%, 12.5%, and 13.5% in mAP50:90 respectively, with Precision increasing by over 12%, demonstrating stronger performance in localization accuracy and false detection suppression. In terms of inference speed, MSRRT-DETR has significantly fewer parameters than most two-stage models, achieving 30.2 FPS – far exceeding Faster R-CNN (6.6 FPS) and Cascade R-CNN (2.1 FPS), indicating excellent real-time capability and deployment potential. In terms of computational cost (GFLOPs), its overhead is only 12.1%–65.5% of that of the two-stage models. Its coordinated design of accuracy, speed, and model size makes it better suited for practical detection requirements in complex orchard scenarios.

Compared to the RT-DETR series, MSRRT-DETR achieves significant improvements in both detection accuracy and inference efficiency. Specifically, versus RT-DETR-50, our method improves mAP50:90 by 2.6 percentage points while more than doubling the inference speed (from 15 FPS to 30.2 FPS), with a computational cost only 49.2% of that of RT-DETR-50. Compared to RT-DETR-L, it achieves a 3.7 percentage point improvement in mAP50:90 with approximately 33.6% faster inference speed and a computational cost only 60.6% of that of RT-DETR-L.

In summary, while maintaining the global modeling capabilities of Transformer, the improved RT-DETR model achieves an optimal balance between detection accuracy and inference efficiency through the introduction of: (1) multi-scale feature extraction module (MSBlock), (2) spatial-channel collaborative attention (SCSA), and (3) efficient feature fusion network (RepGFPN). In addition, statistical significance tests further validate the robustness of the proposed method. For most evaluation metrics, MSRRT-DETR achieves statistically significant improvements over competing models (p < 0.05). For the few cases where no significant difference is observed, the performance gaps are marginal, indicating that MSRRT-DETR maintains comparable accuracy while offering clear advantages in high-precision detection and efficiency.

To facilitate intuitive comparison of each model’s comprehensive performance in accuracy and efficiency, this study introduces the Composite Score as an evaluation metric, calculated as follows:


$$\text{Composite Score }=\;\frac{{\text{map50}}_{\text{i}}-{\text{map50}}_{\text{min}}}{{\text{map50}}_{\text{max}}-{\text{map50}}_{\text{min}}}\;+\;\frac{{\text{FPS}}_{\text{i}}-{\text{FPS}}_{\text{min}}}{{\text{FPS}}_{\text{max}}-{\text{FPS}}_{\text{min}}}$$ (12)

where ${\text{map50}}_{\text{i}}$ represents the mAP50 of the i-th model, and ${\text{FPS}}_{\text{i}}$ represents the FPS of the i-th model.

**Fig 11:**
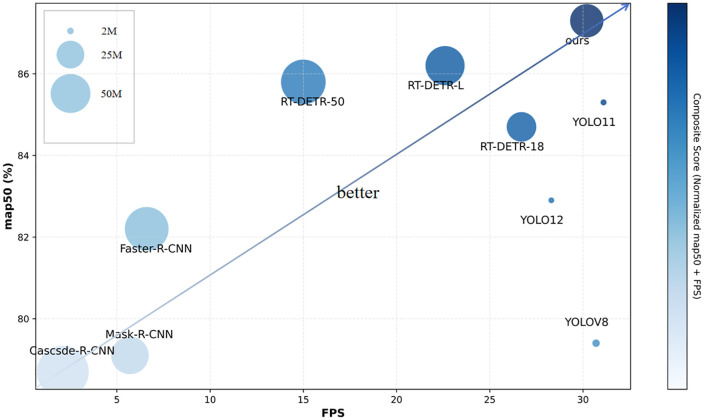
Performance comparison of different object detection models in terms of FPS, mAP50, parameter count and Composite Score. The horizontal axis represents the model’s FPS value, while the vertical axis represents the model’s mAP50 value. The circle size indicates the model’s parameter count (model complexity), with larger circles representing higher parameter counts. The color depth of the circles represents the model’s comprehensive score, where darker colors indicate better overall performance in both accuracy and speed.

As shown in Fig 11, compared to two-stage detectors and YOLO series models, MSRRT-DETR achieves the highest Composite Score (indicated by the darkest color) and is positioned along the diagonal. This demonstrates that its high Composite Score is not due to exceptional performance in one single metric while performing mediocre or poorly in others. These results indicate that MSRRT-DETR successfully achieves an excellent balance across three key dimensions – accuracy, inference speed, and parameter count – demonstrating its significant advantages in comprehensive model performance.

### Cross-domain generalization experiment

**Fig 12:**
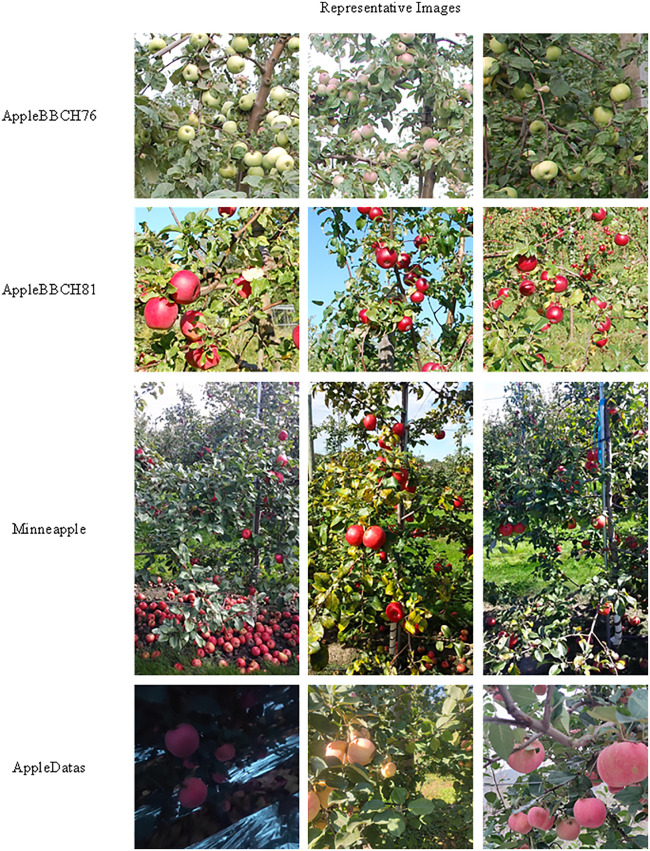
Sample images from each dataset used in the generalization experiments.

To comprehensively evaluate the cross-domain generalization capability of the proposed model, this study selected four apple detection datasets that were not involved in training for validation, covering different fruit maturity stages (AppleBBCH76, AppleBBCH81) [31], complex background and small target scenarios (Minneapple) [32], as well as datasets with significant lighting variations and occlusion interference (AppleDatas). The dataset descriptions and basic information are shown in Table 4 and Fig 12. The comparison subjects include YOLO series models (YOLO11, YOLOv8, YOLO12), two-stage detectors (Faster R-CNN, Mask R-CNN, Cascade R-CNN), RT-DETR series models (RT-DETR-L, RT-DETR-18, RT-DETR-50), and the proposed improved model.

**Table 4:** Detailed statistics and characteristic descriptions of apple datasets for generalization experiment.

Datasets	Dataset characteristic descriptions	Image	Instances
AppleBBCH76	Developing apple fruits (BBCH stages 76–78), covering multiple shooting angles.	3169	42327
AppleBBCH81	Mature apple fruits (BBCH stages 81–85), covering multiple shooting angles.	1838	15309
Minneapple	Mature apple fruits, covering various lighting conditions with relatively small target objects.	331	12285
AppleDatas	Collected from two orchards, the dataset covers various detection scenarios including lighting conditions, viewing angles, occlusions, and dense distributions.	3151	47858

As shown in Table 5, while the YOLO series demonstrates relatively good performance on certain metrics in standard scenarios, its accuracy declines significantly in complex environments. The two-stage detectors exhibit considerable fluctuations across different datasets for certain metrics, indicating insufficient stability. In contrast, our model achieves more balanced performance across various scenarios with stronger generalization capability.

**Table 5:** Cross-domain generalization performance comparison of different detection models across datasets.

Datasets	Models	mAP50/%	mAP50:90/%	Precision/%	Recall/%	F1-Score/%
AppleBBCH76	YOLOv8	64.8	33.7	75.7	55.3	63.9
	YOLO11	70.3	37.8	77.6	59.7	67.5
	YOLO12	65.4	34.5	72.8	56.2	63.4
	Faster-R-CNN	46.8	24.9	82.9	50.0	62.4
	Mask-R-CNN	36.6	20.2	90.1	37.6	53.0
	Cascsde-R-CNN	34.9	21.5	89.5	35.1	50.4
	RT-DETR-L	69.6	36.8	74.9	61.2	67.4
	RT-DETR-18	70.3	37.9	75.4	61.3	67.6
	RT-DETR-50	75.8	41.1	81.2	66.2	72.9
	MSRRT-DETR (ours)	71.9	38.9	79.3	62.3	69.8
AppleBBCH81	YOLOv8	75.1	42.5	82.3	64.3	72.2
	YOLO11	79.7	45.7	84.0	68.2	75.3
	YOLO12	73.9	42.3	78.9	63.2	70.2
	Faster-R-CNN	66.6	44.1	73.8	69.9	71.8
	Mask-R-CNN	65.7	35.6	85.5	67.9	75.7
	Cascsde-R-CNN	53.3	34.8	79.5	56.6	66.1
	RT-DETR-L	77.8	44.3	81.3	68.6	74.4
	RT-DETR-18	79.0	45.2	82.2	68.9	75.0
	RT-DETR-50	82.4	47.3	86.3	72.1	78.6
	MSRRT-DETR (ours)	82.5	47.5	87.9	72.1	79.2
Minneapple	YOLOv8	35.8	14.6	45.2	36.5	40.4
	YOLO11	35.8	15.9	44.8	36.8	40.4
	YOLO12	34.4	13.1	42.1	37.4	39.6
	Faster-R-CNN	27.7	13.7	63.4	32.7	43.2
	Mask-R-CNN	14.0	6.3	63.6	16.6	26.4
	Cascsde-R-CNN	12.8	5.8	70.1	14.4	23.9
	RT-DETR-L	36.8	16.2	45.1	40.1	42.5
	RT-DETR-18	32.8	14.5	39.2	39.3	39.2
	RT-DETR-50	35.8	17.5	43.6	38.1	40.7
	MSRRT-DETR (ours)	40.0	19.5	46.0	42.0	43.9
AppleData	YOLOv8	55.8	29.9	71.0	46.8	56.4
	YOLO11	57.5	31.5	71.7	48.0	57.5
	YOLO12	55.0	29.6	67.7	46.3	55.0
	Faster-R-CNN	48.8	26.7	69.4	53.0	60.1
	Mask-R-CNN	43.6	24.3	79.8	46.1	58.4
	Cascsde-R-CNN	61.4	37.7	76.0	64.8	69.9
	RT-DETR-L	60.6	32.8	73.1	52.6	61.2
	RT-DETR-18	59.6	32.5	74.1	51.0	60.4
	RT-DETR-50	63.2	34.8	76.1	54.6	63.6
	MSRRT-DETR (ours)	62.4	34.5	76.1	53.6	62.9

Notably, our model delivers excellent performance across all datasets, particularly achieving the best overall F1-Score of 0.792 on AppleBBCH81. Even in the Minneapple dataset where overall accuracy generally decreases, our model still maintains a leading score of 0.439, demonstrating robust small-target detection capabilities.

**Fig 13:**
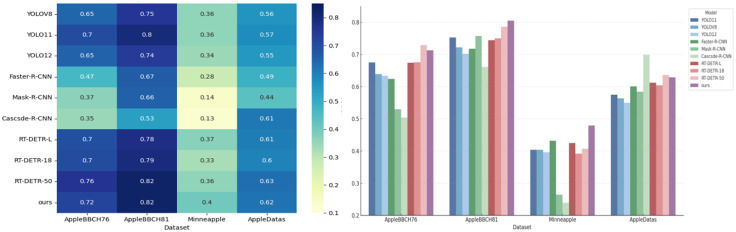
Performance comparison of various detection models on multi-source apple detection datasets (F1-score bar chart and mAP50 heatmap).

As shown in Fig 13, the models demonstrate good overall performance under standard detection conditions such as AppleBBCH76 and AppleBBCH81, but exhibit a general decline in detection accuracy in datasets with dense small targets and complex lighting conditions like Minneapple and AppleDatas, indicating that the robustness of current mainstream detection models in complex scenarios still needs improvement.

Notably, the RE-DETR series models perform significantly better than both the YOLO series and two-stage detectors across all datasets. In particular, both RT-DETR-50 and our model achieve mAP50 scores exceeding 0.82 in the AppleBBCH81 dataset, ranking first among all models. In the Minneapple dataset, although the overall accuracy level decreases significantly, the Transformer series still maintains a relative advantage, demonstrating its modeling capability and cross-domain generalization performance in handling complex environments with small targets and heavy occlusion.

## Conclusion

This paper presents MSRRT-DETR, an end-to-end fruit detection model designed to meet the demands of accuracy, real-time performance, and cross-domain generalization in complex orchard environments. The proposed model integrates a hierarchical multi-scale feature extraction module (MSBlock), a spatial–channel collaborative attention mechanism (SCSA), and an efficient re-parameterized feature fusion neck (RepGFPN), significantly enhancing detection robustness for small, occluded, and low-contrast targets.

Comprehensive experiments demonstrate that MSRRT-DETR achieves superior detection accuracy, fast inference speed (30.2 FPS), and strong cross-domain generalization without the need for retraining. Compared with state-of-the-art YOLO series models, two-stage detectors, and original RT-DETR variants, MSRRT-DETR exhibits notable advantages in both detection precision and computational efficiency.

Despite its overall superior performance, MSRRT-DETR still exhibits limitations in generalization when there is a significant discrepancy between the training data and the target apple varieties. Future research will focus on enhancing model robustness under extreme conditions, including the integration of multimodal data sources (e.g., depth maps or thermal imagery), and the adoption of model sparsification or knowledge distillation techniques to further improve cross-domain generalization and lightweight deployment. These efforts aim to support the continued advancement of intelligent fruit detection systems in complex and dynamic agricultural environments.
